# Time-Kill Kinetics and In Vitro Antifungal Susceptibility of Non-*fumigatus Aspergillus* Species Isolated from Patients with Ocular Mycoses

**DOI:** 10.1007/s11046-015-9969-z

**Published:** 2015-11-26

**Authors:** Yasemin Öz, Havva Gül Özdemir, Egemen Gökbolat, Nuri Kiraz, Macit Ilkit, Seyedmojtaba Seyedmousavi

**Affiliations:** Division of Mycology, Department of Microbiology, Faculty of Medicine, University of Eskisehir Osmangazi, Eskisehir, Turkey; Division of Ophthalmology, Medicosocial Health Center, University of Çukurova, Adana, Turkey; Department of Microbiology, Faculty of Medicine, University of Eskisehir Osmangazi, Eskisehir, Turkey; Division of Mycology, Department of Microbiology, Cerrahpasa Faculty of Medicine, University of Istanbul, Istanbul, Turkey; Division of Mycology, Department of Microbiology, Faculty of Medicine, University of Çukurova, Adana, Turkey; Department of Medical Microbiology and Infectious Diseases, ErasmusMC, P.O. Box 2040, 3000 CA Rotterdam, The Netherlands; Department of Medical Microbiology, Radboud UMC, Nijmegen, The Netherlands; Invasive Fungi Research Center, Mazandaran University of Medical Sciences, Sari, Iran

**Keywords:** Non-*fumigatusAspergillus* spp., Ophthalmic mycoses, Antifungal susceptibility testing, Time-kill assay

## Abstract

*Aspergillus* species can cause ocular morbidity and blindness, and thus, appropriate antifungal therapy is needed. We investigated the in vitro activity of itraconazole, voriconazole, posaconazole, caspofungin, anidulafungin, and amphotericin B against 14 *Aspergillus* isolates obtained from patients with ocular mycoses, using the CLSI reference broth microdilution methodology. In addition, time-kill assays were performed, exposing each isolate separately to 1-, 4-, and 16-fold concentrations above the minimum inhibitory concentration (MIC) of each antifungal agent. A sigmoid maximum-effect (*E*_max_) model was used to fit the time-kill curve data. The drug effect was further evaluated by measuring an increase/decrease in the killing rate of the tested isolates. The MICs of amphotericin B, itraconazole, voriconazole, and posaconazole were 0.5–1.0, 1.0, 0.5–1.0, and 0.25 µg/ml for *A*. *brasiliensis*, *A*. *niger*, and *A*. *tubingensis* isolates, respectively, and 2.0–4.0, 0.5, 1.0 for *A*. *flavus,* and 0.12–0.25 µg/ml for *A*. *nomius* isolates, respectively. *A*. *calidoustus* had the highest MIC range for the azoles (4.0–16.0 µg/ml) among all isolates tested. The minimum effective concentrations of caspofungin and anidulafungin were ≤0.03–0.5 µg/ml and ≤0.03 µg/ml for all isolates, respectively. Posaconazole demonstrated maximal killing rates (*E*_max_ = 0.63 h^−1^, *r*^2^ = 0.71) against 14 ocular *Aspergillus* isolates, followed by amphotericin B (*E*_max_ = 0.39 h^−1^, *r*^2^ = 0.87), voriconazole (*E*_max_ = 0.35 h^−1^, *r*^2^ = 0.098), and itraconazole (*E*_max_ = 0.01 h^−1^, *r*^2^ = 0.98). Overall, the antifungal susceptibility of the non-*fumigatusAspergillus* isolates tested was species and antifungal agent dependent. Analysis of the kinetic growth assays, along with consideration of the killing rates, revealed that posaconazole was the most effective antifungal against all of the isolates.

## Introduction

Fungal infections of the eye are still an important cause of ocular morbidity, particularly in developing countries [1]. Ocular mycoses are difficult to treat successfully because many fungal genera and species have been implicated in ocular infections. Notably, it is difficult to choose an appropriate treatment empirically because drugs are typically selected without considering the susceptibility data [2, 3]. Moreover, surgical intervention such as penetrating keratoplasty may be required for severe mycotic keratitis that cannot be treated because of the prognosis of empty lacunae, indicating bone necrosis [4, 5]. Thus, effective treatment with available antifungal agents is important to improve the outcome of ocular mycoses, and therefore, susceptibility testing may help to guide therapeutic decisions if performed in a timely manner.

In general, polyenes are effective against both filamentous and yeast forms of fungi. Natamycin is the only commercially available topical ophthalmic antifungal agent with a broad spectrum of activity against filamentous organisms, particularly for infections caused by *Fusarium*. However, because of poor ocular penetration, it has primarily been useful in cases with superficial corneal infection. In addition, topical amphotericin B is the drug of choice to treat patients with mycotic keratitis caused by yeasts [6].

Topical 1 % voriconazole has been found to be safe and effective for the primary management of mycotic keratitis, with an efficacy matching that of conventional natamycin [7]. In addition, Matsumoto et al. [8] showed that topical 0.1 % micafungin eyedrops are comparable to azoles in the treatment of mycotic keratitis, regardless of the patient’s age, sex, or ulcer size. Various fungal genera, including *Fusarium*, *Aspergillus*, *Candida*, *Curvularia*, *Scedosporium* spp., and *Schizophyllum commune,* frequently infect ocular structures [9–11]. Although most of the *Aspergillus* ocular infections are caused by *A*. *fumigatus*, mycotic keratitis caused by non-*fumigatus Aspergillus* species has increased significantly over the past few years [4]. In a study focusing on the *Aspergillus* genus as a causative agent of mycotic keratitis, *A*. *flavus* was found to be the predominant species (75 %), followed by *A*. *fumigatus* and *A*. *terreus,* as determined by molecular identification [12]. In addition, recent molecular studies revealed that the spectrum of *Aspergillus* species causing mycotic keratitis is much broader than previously believed and includes *A*. *pseudotamarii*, *A*. *tamarii*, *A*. *nomius*, *A*. *tubingensis*, and *A*. *brasiliensis* [13–17].

In the present study, we investigated the in vitro activity of itraconazole, voriconazole, posaconazole, caspofungin, anidulafungin, and amphotericin B against *Aspergillus* species isolated from the infected eyes of different patients throughout the world. The microdilution susceptibility test was performed according to the guidelines of the Clinical and Laboratory Standards Institute (CLSI) [18]. In addition, a microbroth kinetic growth assay was performed to generate basic pharmacodynamic information on the relationship between the various concentrations of the antifungals and the killing rate of each isolate, according to a maximum-effect (*E*_max_) model, as described previously [19, 20].

## Materials and Methods

### *Aspergillus* Isolates

In total, 14 isolates from different clinical sources were obtained from the reference culture collection of the CBS-KNAW Fungal Biodiversity Centre, Utrecht, the Netherlands. Table 1 shows the origin, identification number, and clinical data of each isolate used in the current study.

**Table 1:** Origins and characteristics of *Aspergillus* isolates tested in this study

Species	CBS no.	Substrate of isolation	Country
*Aspergillus brasiliensis*	122723	Corneal ulcer, human	India
*A. brasiliensis*	122724	Corneal ulcer, human	India
*A. calidoustus*	121609	Post-cataract surgery, endophthalmitis	Ankara, Turkey
*A. calidoustus*	121610	Post-cataract surgery, endophthalmitis	Ankara, Turkey
*A. flavus*	126857	Keratitis	Sao Paulo, Brazil
*A. flavus*	126858	Keratitis	Sao Paulo, Brazil
*A. flavus*	616.94	Orbita tumor, human	Aachen, Germany
*A. niger*	122720	Corneal ulcer, keratitis	India
*A. niger*	122721	Keratitis	India
*A. niger*	122722	Keratitis	India
*A. nomius*	123901	Corneal scraping	India
*A. tamarii*	121598	Keratitis	India
*A. tubingensis*	122719	Corneal ulcer, keratitis	India
*A. tubingensis*	122725	Corneal ulcer, keratitis	India

### Antifungal Drugs

Itraconazole (Janssen, Beerse, Belgium), voriconazole (Pfizer, New York, NY, USA), posaconazole (Schering-Plough, Kenilworth, NJ, USA), caspofungin (Merck, Rahway, NJ, USA), anidulafungin (Pfizer, New York, NY, USA), and amphotericin B (Sigma, St. Louis, MO, USA) were obtained as standard powders from their manufacturers. Antifungal stock solutions were prepared in dimethyl sulfoxide (for itraconazole, voriconazole, posaconazole, anidulafungin, and amphotericin B) or distilled water (for caspofungin). The drug dilutions were prepared in RPMI 1640 medium (Sigma-Aldrich, Steinheim, Germany) buffered to pH 7.0 with 0.165 M MOPS (morpholinopropanesulfonic acid; Sigma-Aldrich, Steinheim, Germany). In vitro antifungal susceptibility testing was performed with the six antifungal compounds using a broth microdilution format according to the CLSI guidelines [18], utilizing an XTT dye [19, 21]. The final concentrations of amphotericin B (AMB), itraconazole (ITC), voriconazole (VRC), posaconazole (POS), caspofungin (CAS), and anidulafungin (AFG) ranged from 0.0313 to 16 µg/ml. The solutions were dispensed into 96-well microtiter plates and stored at −70 °C until use. The results were read using a reading mirror and a microtitration plate spectrophotometric reader (BIO-TEK, ELX800, USA).

### Antifungal Susceptibility Testing

Each isolate was subcultured on potato dextrose agar (Merck, Darmstadt, Germany) for 7 days at 35 °C to ensure its viability and purity. The fungal colonies were covered with 1 ml of sterile 0.85 % saline, and a suspension was prepared by gentle probing of the colonies. The resulting suspensions were withdrawn and transferred to a sterile tube where the heavy particles were allowed to settle for 3–5 min, and the upper homogeneous suspension was transferred to another sterile tube. The final fungal suspensions were adjusted to an optical density (OD) of 0.09–0.13 at 530 nm using a spectrophotometer. These suspensions were diluted 1:50 in RPMI 1640, and the final inoculum size was approximately 0.4–5 × 10^4^ CFU/ml. The final inoculum size was also confirmed with a quantitative colony count on Sabouraud glucose agar (Merck, Darmstadt, Germany). Each well of the microtiter plates was inoculated with 0.1 ml of fungal suspension and incubated at 35 °C, and the minimal inhibitory concentrations (MICs) were determined at 48 h. Growth (drug-free) and sterilized (microorganism-free) control wells were included for each isolate, and all of the strains were evaluated twice for antifungal drug susceptibility.

The MIC endpoints for the azoles and AMB were defined as the lowest concentration that produced complete inhibition of growth. The minimum effective concentration (MEC) of CAS and AFG was defined as the lowest concentration at which the fungi display microscopic morphological changes [18].

The quality control strains *Paecilomyces variotii* (ATCC 22319), *Candida parapsilosis* (ATCC 22019), and *Candida krusei* (ATCC 6258) were used in all experiments. The ranges and the geometric means (GM) of the MIC and MEC were determined for each species and drug after 48 h of incubation. The MIC_50_ and MIC_90_ values were not calculated due to the limited number of species. If the MIC value of the replicates was different, the GM values of the replicates were used for comparison with other isolates. All experiments were performed in triplicate on different days.

### XTT and Menadione

XTT (Sigma-Aldrich, St. Louis, MO, USA) was dissolved in saline at a concentration of 1 mg/ml, and after it was completely dissolved, the solution was filtered through a filter with a pore size of 0.22 µm. Menadione (Sigma-Aldrich, St. Louis, MO, USA) was first dissolved in acetone at a concentration of 10 mM and then diluted 1:10 in saline. The final solution of 1 mg/ml XTT with 125 µM menadione was prepared in saline [19, 21].

### Colorimetric Microbroth Kinetic Growth Assay

Killing curves were obtained by following a procedure described previously [20, 22]. Briefly, concentrations 1-, 4-, and 16-fold higher than the determined MIC were calculated for each antifungal agent. Separate microplates were used for each time point of incubation (2, 6, 12, 24, and 48 h). The wells in the first column included 100 µl of media without drugs (growth control), and the wells in the fifth column included 200 µl of media without drugs and conidia (negative control) in each microplate, which were processed in the same manner as the inoculated wells. The next three columns were inoculated with 100 µl of three different concentrations of ITC, VRC, and AMB or CAS, AFG, and POS, with the ninth column left empty. The inoculum suspensions were prepared at a concentration of 1 × 10^6^ to 5 × 10^6^ conidia/ml and diluted 1:5 in RPMI 1640. The inoculum suspension (100 µl) was then inoculated into each well, and all plates were incubated at 37 °C for up to 48 h. Each plate was taken from the incubator 2 h prior to the end of the incubation time, and 50 µl of XTT–menadione solution was added to each well. After 2 h of incubation with XTT, the formazan absorbance for each well was read at 450 and 492 nm with a microplate reader. All experiments were performed in duplicate on two different days. The results were evaluated by comparing the absorbance in the growth with the negative control conditions.

### Curve Fitting and Analysis

The experimental data derived from the colorimetric growth assays were analyzed using GraphPad Prism 5.3 (GraphPad Inc., San Diego, CA, USA) as described previously [22–24]. Log-transformed optical densities were plotted against time for each antifungal agent. The kill rate was determined at time intervals of 2–48 h for the azoles and AMB and 2–24 h for the echinocandins via a linear regression analysis. The log-transformed concentrations were then plotted against the slopes obtained from the linear regression analysis of the log-transformed optical densities versus time for each antifungal agent against the 14 tested isolates. The sigmoid maximum-effect (*E*_max_: model four-parameter Hill’s equation) model was used to fit the kill rate data to determine the pharmacodynamic relationship between the antifungal concentration and fungal growth or death. *E*_max_, the 50 % effective concentration (EC_50_), was calculated for each assay. The goodness of fit for the *E*_max_ model was checked by *R*^2^ and visual inspection. The *P* value of <0.05 (two tailed) was considered for statistical significance.

## Results

The antifungal susceptibility test results for all isolates are presented in Table 2. Overall, our results indicated that MIC and MEC endpoints determined by either visual or spectrophotometric readings were similar. The MIC values of AMB were from 0.5 to 4.0 µg/ml, and the lowest MIC values were for *A*. *brasiliensis* and *A*. *niger* isolates, whereas the highest MIC value was for the *A*. *flavus* isolate.

**Table 2:** Geometric mean MICs obtained by susceptibility testing of six antifungal agents

Species	CBS no.	MIC/MEC results (µg/ml)					
		ITC	VRC	POS	CAS	AFG	AMB
*A. brasiliensis*	122723	1.0	0.5	0.25	≤0.03	≤0.03	1.0
*A. brasiliensis*	122724	1.0	1.0	0.25	0.12	≤0.03	0.5
*A. calidoustus*	121609	4.0	16.0	8.0	0.5	≤0.03	2.0
*A. calidoustus*	121610	4.0	8.0	8.0	0.5	≤0.03	2.0
*A. flavus*	126857	0.5	1.0	0.25	0.25	≤0.03	2.0
*A. flavus*	126858	0.5	1.0	0.12	0.25	≤0.03	2.0
*A. flavus*	616.94	0.5	1.0	0.25	0.12	≤0.03	4.0
*A. niger*	122720	1.0	0.5	0.25	≤0.03	≤0.03	0.5
*A. niger*	122721	1.0	0.5	0.25	0.12	≤0.03	1.0
*A. niger*	122722	1.0	0.5	0.25	0.12	≤0.03	0.5
*A. nomius*	123901	0. 5	1.0	0.25	0.12	≤0.03	2.0
*A. tamarii*	121598	0.25	0.5	0.12	0.25	≤0.03	1.0
*A. tubingensis*	122719	1.0	1.0	0.25	0.12	≤0.03	1.0
*A. tubingensis*	122725	1.0	1.0	0.25	0.12	≤0.03	1.0
*P. variotii* (QC)	ATCC 22319	0.12	0.06	0.06	≤0.015	≤0.015	2.0
*C. parapsilosis* (QC)	ATCC 22019	0.5	0.12	0.12	1.0	1.0	1.0
*C. krusei* (QC)	ATCC 6258	0.25	0.25	0.12	1.0	0.06	2.0

*ITC* itraconazole, *VRC* voriconazole, *POS* posaconazole, *CAS* caspofungin, *AFG* anidulafungin, *AMB* amphotericin B, *QC* quality control

ITC MIC values were 0.25 µg/ml for *A*. *tamarii*, 4.0 µg/ml for *A*. *calidoustus* isolates, and 0.5–1 µg/ml for the other isolates. The MIC values of VRC were 0.5–1 µg/ml for all isolates except for *A*. *calidoustus*, which had the highest MIC range for azoles (4.0–16.0 µg/ml) among all of the isolates tested. The lowest azole MICs were observed with POS; except for *A*. *calidoustus*, all MIC values of POS were 0.12–0.25 µg/ml. The MEC values of echinocandins were very low; all AFG MECs were ≤0.03 µg/ml, and the CAS MECs were ≤0.03–0.5 µg/ml. In addition, the MIC/MEC values of the drugs tested on the quality control isolates were in acceptable ranges (Table 2).

**Fig. 1:**
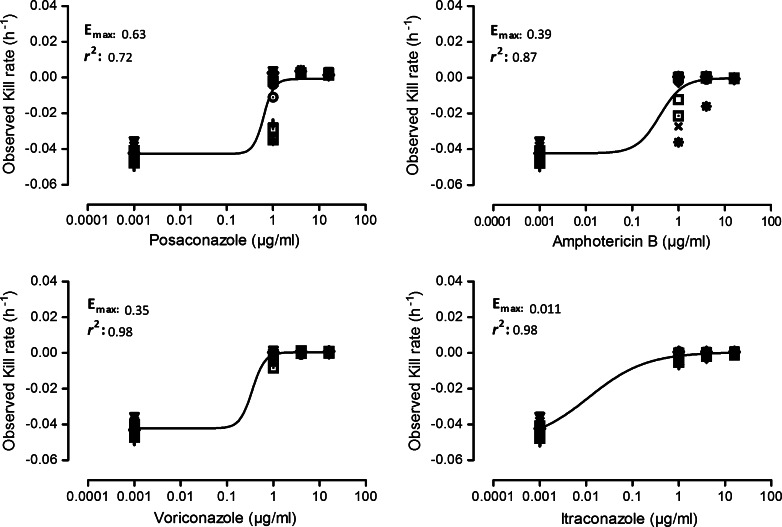
Best-fit sigmoid curves obtained from the *E*_max_ model of non-*fumigatus Aspergillus* species exposed to various antifungals. Posaconazole had the highest *E*_max_ (0.63 h^−1^, *r* ^2^ = 0.71), followed by amphotericin B (0.39 h^−1^, *r* ^2^ = 0.87), voriconazole (0.35 h^−1^, *r* ^2^ = 0.098), and itraconazole (0.01 h^−1^, *r* ^2^ = 0.98). The kill rate was determined at time intervals of 2–48 h via a linear regression analysis. The sigmoid maximum-effect model was then used to fit the time-kill curve data. The symbols shown represent slopes obtained from linear regression analysis of log-transformed optical densities versus time for each antifungal agent against 14 isolates

Figure 1 shows the relationship between the kill rate and the concentration for all *Aspergillus* species evaluated. The *E*_max_ model was a good fit for the kill rate data. Posaconazole had the maximal killing rates (*E*_max_ = 0.63 h^−1^, *r*^2^ = 0.71) against the 14 ocular *Aspergillus* isolates, followed by amphotericin B (*E*_max_ = 0.39 h^−1^, *r*^2^ = 0.87), voriconazole (*E*_max_ = 0.35 h^−1^, *r*^2^ = 0.098), and itraconazole (*E*_max_ = 0.01 h^−1^, *r*^2^ = 0.98), indicating that posaconazole was the most effective antifungal agent against all of the isolates tested. However, it was not possible to determine the maximal and half-maximum killing rates for CAS and AFG against any of the non-*fumigatusAspergillus* species tested.

## Discussion

Ocular mycoses are serious infections of the cornea, orbit, and other ocular structures that may result in blindness or reduced vision [11]. Successful therapy for ocular mycoses relies on identification of the causal agents at the species level and antifungal susceptibility testing. Importantly, the in vitro susceptibility testing of fungi isolated from ophthalmic lesions is considered to guide the clinician in the selection of an appropriate antifungal compound [4].

The pattern of growth and killing rates of all isolates were further investigated by a microbroth kinetic growth assay. Both azoles and AMB were the most potent antifungal agents against all isolates tested; in contrast, echinocandins did not completely inhibit the growth of any of the tested isolates in a concentration-dependent manner.

Recent revisions of the taxonomy of *Aspergillus* spp. have had major implications for the understanding of drug susceptibility profiles [23]. New sibling species of *A*. *fumigatus* exhibit in vitro susceptibility profiles that differ significantly from that of *A*. *fumigatus*. Whereas acquired azole resistance is an emerging problem for *A*. *fumigatus* [24, 25], other *Aspergillus* spp. may be intrinsically resistant to specific classes of antifungal agents (Table 2). The MIC of AMB and azoles for some of the non-*fumigatus Aspergillus* spp. was elevated compared with those for *A*. *fumigatus* [23]. The MIC of AMB in *A*. *flavus* clinical isolates was consistently two-fold higher than that in *A*. *fumigatus* [26]. Using CLSI, AMB was shown to have MIC values of 1–2 mg/l in *A*. *nidulans*, which was higher than the values commonly observed with *A*. *fumigatus* [27]. In the section *Usti*, the azoles were not active against *A*. *calidoustus,* with MICs of ≥8 mg/l, and the other classes of antifungal drugs also appeared to be less active compared with their activity against *A*. *fumigatus*. For instance, the MICs of AMB were shown to be 1–2 mg/l, which is relatively high [28]. The resistance of *A*. *terreus* to amphotericin B is well recognized [29]. Based on azole susceptibility, three different susceptibility patterns were distinguished in the black aspergilla, *Aspergillus*, section *Nigri*. Azoles showed low MICs in some isolates and high MICs in other isolates; a third group of isolates showed an uncommon paradoxical effect. However, these groups did not coincide with species boundaries, making it difficult to interpret the difference in MIC as an intrinsic or acquired property of these molds [30, 31].

In general, voriconazole is the recommended first-choice drug for the prophylaxis and management of mycotic endophthalmitis and keratitis against filamentous organisms [32, 33]. However, new-generation triazoles such as posaconazole have shown good safety profiles in both laboratory and clinical studies [34]. In the current study, we also found that posaconazole had a lower MIC than voriconazole against all non-*fumigatus Aspergillus* species except *A*. *calidoustus* isolates, which had the highest MIC values (≥4.0 µg/ml) against all azoles.

The growth and kill curves of POS did not show apparent inhibition, while ITC and VRC exhibited persistent inhibition against *A*. *calidoustus* isolates at concentrations equal to or higher than the MIC. This newly described isolate is considered to be a relatively rare human pathogen but appears to represent an emerging problem, as *A*. *calidoustus* isolates have been found to be triazole resistant in several recent studies [28, 35–37]. Although our findings reaffirm the elevated triazole MIC for *A*. *calidoustus* isolates, the POS resistance was more evident when using the time-kill method with the XTT colorimetric assay in our in vitro study. ITC and VRC exhibited prominent inhibition at concentrations equal to or higher than the MIC, even though the MIC was elevated. In this scenario, topical usage of ITC and VRC at higher doses may be promising for treatment of ocular infections caused by *A*. *calidoustus*. However, variation in the activity of the itraconazole should be considered depending on the type of *Aspergillus* species. In studies reported by an Indo-Hungarian group, itraconazole showed a higher in vitro MIC in *A. tubingensis* isolates obtained from mycotic keratitis [12, 16].

CAS and AFG had the lowest MEC values against all of the *Aspergillus* isolates tested; however, they did not exhibit concentration-dependent inhibition of fungal growth in the time-kill assay, possibly because of the heterogeneous growth characteristics of *Aspergillus* spp. [21] and the paradoxical effects of echinocandins [38]. In several recent in vitro studies incorporating ocular isolates, CAS and AFG presented excellent activity against various *Aspergillus* species in the broth microdilution method, in which the MEC values were low (≤0.008–1 µg/ml for CAS and ≤0.001–0.015 µg/ml for AFG) [2, 39–42]. Similar to our study, Lockhart et al. determined the echinocandin MEC values for caspofungin, micafungin, and anidulafungin against 288 *Aspergillus* isolates prospectively collected from transplant patients [43]. The MEC ranges for *A*. *flavus*, *A*. *niger*, *A*. *calidoustus*, and *A*. *tubingensis* were, respectively: 0.008–0.03 µg/ml for CAS and 0.008–0.015 µg/ml for AFG, 0.015–0.5 µg/ml for CAS and 0.008–0.03 µg/ml for AFG, 0.03–4 µg/ml for CAS and 0.008–0.06 µg/ml for AFG, and 0.015–0.03 µg/ml for CAS and 0.008 µg/ml for AFG [43].

In comparison with the other antifungal agents tested, we observed that the MIC of AMB tended to be higher against various *Aspergillus* species, which is similar to the findings reported in other studies including both ocular [2, 11, 44] and non-ocular isolates [28, 35, 42], with MIC values of ≤4 µg/ml. In our study, the time-killing assay of AMB demonstrated complete concentration-dependent inhibition of the growth of all isolates.

Of note, as discussed above, the results of the time-kill studies performed in the current study provided a more dynamic assessment of the interaction between the antifungals and the fungi isolated from ocular lesions than the static MIC determinations did. This indicates that the time-kill assay may have a greater clinical utility for guiding therapy in an individual patient. However, it should be noted that in the clinical setting, standardized in vitro MIC studies are easier to perform than monitoring of fungal growth in the time-kill kinetic system.

Furthermore, for an adequate therapeutic response, in addition to choosing an antifungal drug with a high level of activity against the etiological ocular pathogen, the drug must be non-irritating and non-toxic to the eye and penetrate well through the corneal layers [45]. Depending on the etiologic agent and the location and extent of the infection in the eye, various antifungal agents and several routes of administration, including intravenous, oral, topical, subconjunctival, intrastromal, intracameral, and intravitreal, should be considered [33]. Natamycin is the only commercially available topical antifungal agent approved by the US Food and Drug Administration (FDA) for ophthalmic use [33, 46]. Alternatively, topical administration of econazole, voriconazole, and posaconazole is recommended for the treatment of mycotic keratitis caused by molds [47].

However, for systemic treatment, the pharmacokinetic variability of the selected antifungal must be considered [48]. Amphotericin B has poor ocular penetration after intravenous (IV) administration and is known to cause severe renal toxicity [33, 49]. Penetration of itraconazole into the eyes is typically insufficient [50]. In contrast, the newer triazoles voriconazole and posaconazole are highly bioavailable and demonstrate good penetration into different parts of the eye [33].

In conclusion, our data contribute to a better understanding of the activity of antifungals used for treatment of disease caused by non-*fumigatusAspergillus* species. Given the limited clinical evidence to support the treatment of non-*fumigatus Aspergillus* species, antifungal susceptibility testing may help to guide therapy, if performed in a timely manner. The colorimetric microbroth growth assay may also provide basic information on the individual effect of static concentrations of each antifungal on these fungi. A major limitation of the present study was the relatively low number of strains studied. Clearly, a wider range of isolates should be studied before any generalizations can be made. In addition, further dynamic in vivo modeling and clinical studies are required to investigate the correlation between in vitro susceptibility and in vivo clinical results.
